# Structural insights into the exosite-mediated activation of factor IX by factor XIa using cryogenic electron microscopy

**DOI:** 10.1016/j.jtha.2026.08.015

**Published:** 2026-08-22

**Authors:** Bassem M. Mohammed, Samantha Deavila, Tristan Friet, Isabella Dattilio

**Affiliations:** Edward A. Doisy Department of Biochemistry and Molecular Biology, Saint Louis University School of Medicine, St. Louis, MO 63104, USA

**Keywords:** cryo-electron microscopy, factor IX, factor XI, hemophilia

## Abstract

**Background:**

Factor (F)XI occupies a unique and clinically significant niche, bridging the tissue factor–driven and contact-driven coagulation pathways. Irrespective of the trigger, activated factor XI (FXIa) contributes to clotting by activating FIX. Biochemical studies established that this reaction requires the membrane-binding FIX-Gla domain to engage an exosite on the FXIa Apple 3 (A3) domain, which only becomes available upon FXI activation. Structural data for FXIa, FIX, FIXaβ and the FXIa:FIX complex are lacking; current understanding relies on zymogen FXI crystal structures and homology modeling of FXIa after kallikrein.

**Objectives:**

Elucidate the high-resolution structure of FXIa in a functionally relevant conformation in complex with FIX.

**Methods:**

We utilized cryo-electron microscopy to determine the structures of human FXIa in complex with its full-length substrate, FIX, and activated product, FIXaβ.

**Results:**

We report the first cryo-electron microscopy structures of FXIa in complex with its substrate, FIX, and activated FIX (FIXaβ). The structures capture a functionally relevant conformational change in the FXIa catalytic domain and reveal the first view of the entire FIX and FIXaβ. Critically, we visualize the FIX-Gla domain precisely docked to the FXIa-A3 exosite on both subunits of the FXIa dimer. We also define the first step of proteolysis, visualizing the FIX Arg145 inserted into the primary specificity pocket of FXIa.

**Conclusions:**

The structures define the full FXIa:FIX interface, providing a structural template for understanding the sequential activation of FIX and for developing a new class of selective allosteric antithrombotic agents.

## 1 | INTRODUCTION

Activated factor XI (FXIa) is the protease form of the plasma zymogen, factor (F)XI. FXI serves a dual role as a clotting and contact factor, bridging contact-driven processes (dispensable for hemostasis) to tissue factor–driven thrombin generation (critical for hemostasis) [1,2]. FXIa contributes to coagulation primarily by activating FIX [1,3]. The 2 factors are differentially important for hemostasis. Congenital FXI deficiency (hemophilia C) presents with a mild bleeding phenotype that is mostly provoked, consistent with a modest hemostatic role, whereas FIX deficiency (hemophilia B) results in severe bleeding, underscoring the crucial role of FIX in hemostasis [4–7]. Given the mild phenotype of hemophilia C, FXIa activation of FIX appears functionally redundant in many contexts, becoming critical mostly in tissues with high fibrinolytic activity, such as mucosal membranes [5]. In these tissues, sustained FIX activation by FXIa stabilizes the forming clot to prevent dislodging and rebleeding. Conversely, high levels of FXI in the general population are associated with increased thrombotic risk, and FXIa generated via contact activation is recognized as the primary driver of thrombosis [4,5]. Consequently, the quest for safer anticoagulants over the last decade has focused on the intrinsic pathway, specifically FXI. Several FXI-targeting agents, including orthosteric small molecules, antisense oligonucleotides, and monoclonal antibodies, are currently being investigated in clinical trials to prevent stroke and venous thromboembolism [4,8–15].

FXI is unique among coagulation proteases in being a homodimer of 2 identical 80-kDa subunits. Each subunit consists of 4 apple domains (A1-A4) forming the heavy chain (HC), followed by a serine protease catalytic domain (CD) that constitutes the light chain (LC) [1]. Crystal structures of zymogen FXI revealed the apple domains to be arranged in a planar disk-like “saucer,” with the CDs sitting atop like a “cup” [16,17]. Dimerization is mediated by an interchain covalent disulfide bond at Cys321 on the A4 domain and numerous noncovalent interactions at the A4 dimer interface [16,17]. The FXI homolog, prekallikrein (PK), is the only other protein known to share this structural architecture, except that PK is a monomer [1,18]. FXI activation involves cleavage of the Arg369 bond within the activation peptide (AP) that lies between the FXIa-HC and FXIa-LC. FXIa-CD remains tethered to the A4 domain via a Cys362–Cys482 disulfide bond [1]. Its primary substrate, FIX, is a vitamin K-dependent zymogen. FIX shares the canonical architecture of this family of clotting factors, starting with an N-terminal membrane-binding Gla domain, followed by 2 epidermal growth factor (EGF)-like domains, an AP, and a CD. Conversion of FIX to the active protease, FIXaβ requires sequential cleavage by FXIa, first at Arg145 to form the intermediate FIXα and subsequently at Arg180 to release AP [19–24].

Biochemical investigation of FIX activation by FXIa has been impeded by the absence of high-resolution structural data for the complex or its full-length components. While crystal structures are available for the zymogen, FXI [16,17], and the homolog active protease, plasma kallikrein (PKa) [17], structures for FXIa, PK, and full-length FIX or FIXaβ are lacking. Consequently, structural models of FXIa historically relied on homology modeling after PKa [17,25]. This approach is limited as FXIa and PKa are functionally distinct, with PKa being a poor activator of FIX. In fact, this functional divergence laid the foundation for early biochemical studies utilizing FXI/PK chimeras [18] and mutagenesis [26] to map the FIX-binding site to an exosite on the FXIa-A3 domain, which becomes exposed only upon FXI activation. Furthermore, the CD of PKa is oriented 180° relative to the PKa apple domain platform as compared with FXI [17]. Recent solution-phase hydrogen–deuterium exchange studies of FXIa suggest that such rotation does not take place in FXIa [27]. Compounding these findings are alternative reports that implicated the FXIa-A2 domain [28] or a secondary exosite on FXIa-CD [29] in substrate recognition. Collectively, these various observations are difficult to reconcile without a structure, leaving the precise topology of the FXIa:FIX interface and the mechanism of sequential processive activation debated.

Herein, we utilized cryo-electron microscopy (cryo-EM) to determine the structure of FXIa in complex with FIX and FIXaβ. The new structures define the full interface, revealing for the first time the FIX-Gla domain docking precisely onto the FXIa-A3 exosite of both subunits of the dimer. This direct visualization provides the long-awaited proof for earlier biochemical studies that established this assembly to be calcium-dependent and requiring the Ω-loop of the FIX-Gla domain [26,30]. Critically, we capture the first step of FIX proteolysis, visualizing FIX-AP with Arg145 seated into the primary specificity pocket of FXIa. We also resolved the final, post-Arg180 cleavage step, within the FXIa:FIXaβ complex, demonstrating that Arg145 provides an additional tether for the mature FIXaβ to FXIa. These findings provide a structural template for understanding FIX activation and designing new classes of selective antithrombotic agents.

## 2 | METHODS

### 2.1 | Materials

Materials obtained commercially were plasma human FIX (HCIX-0040, Prolytix), pDest40 expression vector (#12274015, Invitrogen), Quantifoil R 1.2/1.3, Cu 300 mesh grids (Q350CR1.3, Electron Microscopy Sciences), Quantifoil R 1.2/1.3, Au/C 200 mesh grids (Q2100AR1.3, Electron Microscopy Sciences), UltrAufoil R 1.2/1.3, Au 300 mesh, (Q350AR13A, Electron Microscopy Sciences), and QuikChange Lightning (#210513, Agilent).

### 2.2 | Recombinant FXI production and activation

cDNA for human FXI (NCBI Ref. Seq. NM_000128.3) with an engineered active site serine mutation (Ser557Ala) was modified to add a sequence encoding a 12-amino acid HPC4 tag (EDQVDPRLIDGK) at the C-terminus and cloned into the pDest40 vector for expression in HEK293 cells (CRL 1573, ATCC). Conditioned media were spun down and supplemented with benzamidine (10 mM) and CaCl_2_ (5 mM), then purified by immunoaffinity using the Ca^2+^-dependent monoclonal antibody against HPC4 as described previously [31]. FXI^S557A^ was activated by incubation in 20 mM HEPES pH 7.4, 150 mM NaCl, 5 mM CaCl_2_ with αFXIIa overnight (Supplementary Figure S1) [19].

### 2.3 | Cryo-EM grid preparation and data collection

Recombinant FXIa^S557A^, plasma FIX, and plasma FIXaβ for cryo-EM studies were buffer-exchanged individually into 20 mM HEPES pH 7.4, 150 mM NaCl, 5 mM CaCl_2_ by size-exclusion chromatography using a Superdex 200 increase 10/300 GL column. At the time of freezing, grids were glow-discharged on an EMS GloQube for 20 seconds. The proteins were then mixed at a 1:4 ratio of FXIa:FIX. Three microliters of the complex mixture at 0.1 mg/mL were applied onto each grid and blotted for 4 seconds, then instantaneously plunge-frozen into liquid ethane on an FEI Vitrobot Mark IV. Grids were loaded onto a Glacios cryo-TEM operating at 200 kV equipped with an FEI Falcon IV (4k × 4k) direct electron detector. Exposures were collected from multiple grids under similar conditions as detailed in Protein Data Bank (PDB) deposition entries. A total of 11 188 micro-graphs were collected for the FXIa:FIX complex, and 11 397 micro-graphs were collected for the FXIa:FIXaβ complex. Sixteen percent of the images for FXIa:FIXaβ were collected at 30° tilt. Relevant data collection parameters, refinement, and validation statistics of the cryo-EM electron density maps and models are summarized in Table 1 and Supplementary Table S1.

### 2.4 | Image processing, map calculation, and model building

cryo-EM data processing was performed in CryoSPARC [32] v4.7.1. The final cryo-EM data processing workflow is summarized in Supplementary Figure S2. Briefly, patch-based motion correction and dose weighting of raw movies were carried out using the Patch Motion job type, followed by patch-based CTF estimation. Particle picking using different approaches (manual, blob, template, and Topaz) and iterative two-dimensional (2D) and 3D classification were conducted, and duplicates were removed till a clean particle stack was obtained. Final particle stacks of 1.54 and 1.32 million particles, for FXIa:FIX and FXIa:FIXaβ, respectively, were fed into a combination of *ab initio* and heterogeneous refinement job types to classify particles in 3D space. During analysis, it became apparent quickly that FIX-Gla domain binding rigidifies FXIa-CD, while FIX EGFs and CD domains remain flexible. Consequently, we targeted 3D classes that show 2 well-defined FXIa-CD and visible FIX-Gla domains. Multiclass 3D classification with a focused mask covering a single FXIa subunit and FIX(aβ) was carried out, and results were compared in ChimeraX-1.10 [33]. Similar volumes were pooled together, capturing different FIX conformations relative to a rigid FXIa. Each volume/particle combination was then processed using non-uniform refinement, giving a consensus refinement volume followed by focused refinements using masks around the core FXIa:FIX-Gla, the interface FXIa-CD:FIX, and the entire FXIa:FIX as deemed necessary. Composite maps of the consensus refinement and the respective local refined maps were generated by aligning the individual maps in ChimeraX and taking the maximum value at each voxel. Maps were sharpened using the Deep-EMhancer [34] wrapper in CryoSPARC. Giving the apparent C2 symmetry of the FXIa:FIX complex, an alternative approach to maximize capturing FIX(aβ) conformations was to apply symmetry expansion and subsequent 3D classification with focused refinement of only one FXIa-subunit:FIX. The approach conformed to the rigorous C1 analysis pipeline and did not separate new conformations. This could be explained by the fact that C1-based 3D reconstructions biased the more defined FIX particle to one side of the dimer. Consensus maps, focused maps, masks, and their respective half-maps and models were deposited in the Electron Microscopy Data Bank (EMDB) and PDB. Map resolution was estimated using gold-standard Fourier shell correlation = 0.143. An initial model for the complex was generated using PDB 6I58 [17] and 5Q0D [35] for FXIa, and PDB 1RFN [36], AlphaFold [37] P00740-F1-v6, and 1NL0 [38] for FIX by docking individual domains as rigid bodies using ChimeraX. All atoms were refined, and model building was completed manually in COOT [39]. The final model was refined against the respective map with secondary structure restraints applied in Phenix [40]. Figures were generated using ChimeraX [33].

## 3 | RESULTS

### 3.1 | Structure determination of the FXIa:FIX and the FXIa:FIXaβ complexes

The cryo-EM structure of the FXIa:FIX and the FXIa:FIXaβ complexes were determined at overall resolutions of 3.44 and 3.32 Å, respectively (Figure 1A, B and Supplementary Figure S3A, B, D). The high-fidelity density maps enabled a comprehensive modeling approach that facilitated an unambiguous view of the molecular architecture. For both complexes, the data allowed refinement of the FXIa homodimer side chains using an *ab initio* modeling approach. This level of detail was particularly critical for tracing the continuous density of the interdomain linkers, which defined a unique domain-domain topology for the activated protease, FXIa, compared with its zymogen, FXI.

For the substrate, FIX, the density permitted a combined modeling strategy where the tethered and more rigid FIX-Gla domain was *ab initio* modeled, defining its precise docking at the FXIa-A3 exosite. In the FXIa:FIX complex, the densities for the FIX-EGF domains and AP were best suited for a backbone trace modeling approach (Supplementary Video S1). This allowed capturing of the initial stage of proteolysis, visualizing the FIX AP with Arg145 inside the primary specificity (S1) pocket of FXIa-CD and oriented for the first cleavage event (Figure 1A right panel). The distal FIX CD was incorporated via rigid-body fitting relying on helical secondary structure landmarks, thus completing the first full-length visualization of the FIX zymogen within a functional complex.

For the FXIa:FIXaβ complex, the more ordered FIXa-CD allowed for a full backbone trace of the EGFs and CD (Supplementary Video S2). We clearly visualize the new N-terminus, Val181, inserted into the mature FIXa-CD, the hallmark of serine protease activation (Supplementary Figure S3C and Supplementary Video S3). An unexpected observation in this structure was that FIXaβ remained tethered not only to the FXIa-A3 exosite but also to the CD of FXIa via Arg145 (P1 of the first cleavage site), like FIX. Specifically, Arg145 is visualized inserted deep into the FXIa-CD S1 pocket (Figure 1C right panel). Additionally, the map quality for both complexes permitted the direct visualization of all N-linked glycosylation modifications on FXIa (modeled), and 2 O-linked glycosylation modifications on FIX (not modeled), as detailed in Figure 1.

It is imperative to note that the global architecture of the complex is characterized by an apparent C2 symmetry dictated by the FXIa homodimeric scaffold. During data analysis, it was observed that while the FXIa scaffold and FIX-Gla remain remarkably rigid and symmetrical, the remaining tethered domains of FIX molecules assumed a range of stochastic, flexible conformations hovering over FXIa-CDs, facilitated by FIX interdomain flexible linkers as resolved by 3D-Flex analysis (Supplementary Video S4). Of note, while this analysis characterizes continuous conformational heterogeneity within the resolved particle population, it does not by itself establish that these conformations represent sequential intermediates along a physiological reaction trajectory. Four conformers were separated and modeled (Supplementary Figure S4), including the high-resolution “class 3” corresponding to the main FXIa:FIXaβ complex plus 3 other conformers at lower resolution. Consequently, refinement was focused on a single FXIa-monomer:FIX(aβ) functional unit using C1 symmetry as detailed in the methods. This strategy is justified by the observation that the conformational state of FIX on one protomer does not influence the distal subunit and the FXIa CD are spatially sequestered at a distance sufficient to preclude any intersubunit steric interference or cooperative substrate engagement. This structural analysis-based independence of the subunits is supported by biochemical data demonstrating that the monomeric FXIa variant activates FIX at rates indistinguishable from the wild-type dimer *in vitro* [41]. Analogous isolation of conformers was not possible for FXIa:FIX as we prioritized resolving residues on the P^′^ side of the FIX-AP, a region that is typically delocalized (high B-factor) in crystal structures of catalytically dead serine protease bound to short substrate-mimicking peptides [42–44].

### 3.2 | The FXIa:FIX interface

The high-resolution structure of the FXIa:FIX assemblies provides a framework for the foundational biochemical observations that initially identified the FXIa A3 domain as a critical substrate exosite [18]. Early mutagenesis and competitive inhibition studies highlighted 2 primary clusters in the A3 domain (residues 183–188 and 225–234) as being essential for FIX recruitment, and that Ca^2+^ and the FIX-Gla domain are crucial for the interaction [19,26,30]. The new structures confirm that this assembly is calcium-dependent; the coordination of multiple Ca^2+^ ions rigidifies the FIX-Gla domain, folding it into the precise spatial geometry required for exosite engagement. Furthermore, the new structures visualize the Ω-loop of FIX-Gla, showing that residue Phe9 is strongly sequestered within a shallow hydrophobic pocket formed by Ile183, Phe187, Pro247, Phe192, and Phe260, providing the primary mechanical anchor for high-affinity docking (Figure 2A). This molecular recognition is further stabilized by a 2.74 Å salt bridge between FIX-Gla30 and FXIa-Asp185. Furthermore, Asp185 shares Ca^2+^ coordination with both FIX-Gla26 and FIX-Gla30. Concurrently, polar interactions involving FXIa-Arg250 with FIX-Gly4 (3.12 Å), and nonpolar interactions involving FXIa-Ala181 and FIX-Gln11 (3.62 Å), clamp the C- and N-termini of the Ω -loop of FIX, respectively (Figure 2B). Beyond this primary recruitment, the structure identifies a secondary, unpredictable contact interface between the FIX-Gla domain and the FXIa-CD that could not be predicted by previous models. Specifically, FIX-Gla36 forms a 3.01 to 3.25 Å electrostatic contact with FXIa-CD-His538, while the adjacent FIX-Thr35 is positioned within 2.84 Å of FXIa-CD-Arg489 (Figure 2C). Structurally, these secondary tethering points provide an additional layer of substrate orientation in tandem with the Ala181 and Arg250 clamps, probably compensating for the relatively shallow nature of the A3 hydrophobic pocket. Simultaneously, the structure captures the first step of proteolysis, where FIX-Arg145 (the P1 residue) is seated precisely within the FXIa specificity pocket, anchored by a robust 2.70 Å salt bridge with Asp551 (Asp189)c (Figure 2D). Notably, this architecture extends to the FXIa:FIXaβ complex, where the fundamental docking mechanism remains identical to the zymogen assembly. All primary interactions at the FXIa-A3 exosite, including the Phe9 hydrophobic burial and the Asp185–Gla30 salt bridge, are preserved, as are the newly identified secondary contacts between the Gla domain (Gla36/Thr35) and the FXIa CD (His538/Arg489), in addition to retethering of Arg145 into FXIa-CD (Figure 2E). A complete list of interdomain contacts were extracted using PDBsum [45] and listed in Supplementary Tables S2 and S3.

Analysis of buried surface areas and solvation energy at the interface using PDBePISA [46,47] further supported the nature of interactions described above. The primary affinity driver of the complex is the exosite interface between the FXIa-A3 domain and the FIX-LC, FIX-LC (structurally encompassing the EGF domains, Gla domain, and the AP). This interaction buries ~530 Å^2^ of surface area per molecule with a favorable solvation free energy (Δ *G*) of −4.7 kcal/mol. Anchored by this A3 interaction, the FIX-LC forms an extensive orienting footprint against FXIa-CD, burying 788.7 Å^2^ on FXIa-CD and 897.5 Å^2^ on FIX-LC. However, this broad interface exhibits a weak Δ *G* of −0.6 kcal/mol. This low thermodynamic stability arises because the physical contacts are driven mostly by only a few residues within the FIX-Gla and FIX-AP regions. Lastly, the interface where these 2 CD ultimately meet in the most stable conformer buries ~250 Å^2^ per molecule with a Δ *G* = −0.8 kcal/mol. Given the continuous conformational heterogeneity of the FIX-CD relative to the FXIa-CD captured in our heterogeneity analysis, we regard this contact as transient, and the specific residue-level interactions extracted here as hypothesis-generating rather than a definitive description of a fixed interface, which is also fitting with the modeling approach used.

### 3.3 | FXI activation and divergence from PKa

The transition from the zymogen FXI to the protease FXIa involves a profound conformational change that extends beyond simple proteolytic cleavage. Our cryo-EM structures provide the first direct visualization of FXIa, defining a unique domain-domain topology that diverges significantly from the zymogen. Global alignment of a single subunit (monomer) of FXI and FXIa yielded a remarkably high root-mean-square-deviation (RMSD) of 5.879 Å across 4338 pruned atoms, indicating a massive macroscopic structural transition. To isolate the source of this displacement, we performed localized alignments. Superimposition of the rigid HC platforms yielded a tight Cα RMSD of 0.99 Å over 331 pruned atoms, demonstrating that the Apple domain core remains virtually unchanged. Similarly, aligning the CD independently revealed a highly conserved core with a Cα RMSD of 0.68 Å over 205 pruned atoms. Taken together, these metrics demonstrate that the CD moves essentially as a rigid body without undergoing significant internal structural remodeling. In FXIa, the dimeric Apple domain platform maintains a rigid localized scaffold, while the CD undergoes a conformational change. This shift is essential for exposing the FIX exosite on FXIa-A3 and positioning the FXIa-CD in a functional conformation optimized for substrate engagement (Figure 3A). Notably, this CD conformation remains consistent whether the enzyme is bound to FIX or FIXaβ. The new position of the CD in FXIa relative to the Apple platform is maintained through a hierarchical network of noncovalent polar and hydrophobic interactions. First, direct FXIa-CD Apple domain interactions with A4 and A3 of the FXIa-HC. Second, the new C-terminus of the FXIa-HC (the AP region) acts as a central structural stabilization hub. It occupies the space between the CD, A1, A2, and A3 domains and mediates an extensive network of contacts between CD and the A1-A2 and A3-A4 linkers, effectively stabilizing the CD for or during substrate engagement (Figure 3B and Table 2—in contrast to Supplementary Table S4 for zymogen FXI CD:HC contacts). These structural data validate earlier biochemical and biophysical studies using mutagenesis and hydrogen–deuterium exchange that concluded that the A3 becomes more solvent accessible upon FXI activation and less accessible upon FIX binding [27,41]. However, our data established a pivotal divergence from previous PKa-based homology models. Instead of the proposed 180 ° rotation of the CD relative to the apple domain platform, the FXIa-CDs undergo pivoted tipping toward FXIa-A4 rather than A1, with no significant rotation (Figure 3C). Using ChimeraX, we defined a principal axis passing through the CD in both the FXI and FXIa states that lies nearly parallel to the plane of the apple domains, HC. Measuring the angular displacement between these axes allowed us to calculate a 45.6° pivoting angle (Supplementary Video S5).

### 3.4 | Structural view of cross-reactive material-positive variants at the FXIa:FIX interface

The new high-resolution coordinates provide a framework to elaborate on several clinically significant cross-reactive material-positive (CRM+) missense mutations. It is important to emphasize that our commentary on these residues is purely structural and solely relevant to the FXIa-FIX activation axis. Because FXI is only modestly important for physiological hemostasis, it is highly unlikely that defects restricted to this interaction can, at least alone, explain the unpredictable clinical bleeding phenotype in hemophilia C. For FIX, mutations of clinical significance cannot be attributed solely to a defect in FXIa-mediated activation, as the primary activator of FIX is the complex tissue factor-FVIIa, and its essential role in hemostasis is largely FXI-independent, unless a single mutation disrupts multiple functional pathways simultaneously. Therefore, structural defects that strictly impair the FXIa-FIX axis are more likely to explain attenuated clot formation in the context of pathologic thrombosis through the contact pathway rather than explain clinical bleeding. With this context, we structurally evaluated the FIX Phe9Leu/Ala variants, which present with a mild to moderate bleeding phenotype. These missense mutations directly affect the hydrophobic keel of the FIX-Gla domain, the Ω-loop. The bulky Phe at position 9 is well documented as critical for lipid membrane insertion; our complex structures indicate that it is equally crucial for the FXIa-A3 exosite interaction (Figure 2A), corroborating previous chimeric studies utilizing the homologous FVII-Gla domain [26,30]. The Leu/Ala mutations preserve global domain folding and surface electrostatics but probably destroy exosite docking affinity by using a much shorter hydrophobic side chain in an already shallow pocket. As for the FIX-Lys5 that was previously proposed to play a role in FXIa engagement; the new structures suggest that the backbone of Lys5 participates in a potential polar clamping interaction with Arg250 on the FXIa platform as observed in one of the complexes. This interaction does not seem to be a function of the Lys side chain itself. Aside from FIX activation by FXIa, which is minor for hemostasis, Lys5 serves as a vital multifunctional hub, being essential for calcium-dependent membrane anchoring into phosphatidylserine bilayers and serving as the primary molecular determinant for extravascular sequestration by Type IV Collagen within the vascular basement membrane [48–51].

For FXI, our coordinates clarify the structural basis of the clinical CRM+ mutations Arg184Gly and Asp185Asn. In the zymogen state, Arg184 (located in the A2-A3 linker) coordinates interactions with Asp488 of CD (FXIa-CD) and Ile269 of the A3-A4 linker, whereas Asp185 remains entirely unengaged (Supplementary Table S4). Conversely, when FXIa is engaged with its substrate, Arg184 shifts to coordinate interactions between Leu262 and His267 of the A3-A4 linker, successfully positioning His267 to make direct contact with FIX-Gla33 and stabilize FIX binding (Figure 3B). Arg184 also interacts with Gly488 of the FXIa-CD. Asp185 plays a similarly significant role in the active complex, where it contributes to FIX-Gla binding through direct coordination interactions with both a FIX-bound calcium ion and a FIX-Gla30.

## 4 | DISCUSSION

Factor XI occupies a unique niche, bridging the tissue factor–driven and contact-driven coagulation pathways [1]. From an evolutionary standpoint, FXI arose from a gene duplication of its ancestral homolog, PK [52]. Despite sharing ~58% sequence identity and a similar domain organization, these proteins diverged fundamentally in form and function. PK is monomeric and serves as an essential component of the proinflammatory kallikrein-kinin system to liberate bradykinin, whereas FXI evolved into a covalent homodimer with a role in host hemostasis. FXIa contributes to clot formation through the activation of FIX, its primary physiological substrate [1,3]. This evolutionary specialization provided FXIa with unique structural prerequisites for efficient activation of FIX, composed of an unmatched high-affinity exosite for FIX recruitment on A3, and a CD topology optimized for FIX-AP processing.

Biochemical investigation of FIX activation by FXIa has reached a halt due to a lack of high-resolution structural data. While crystal structures exist for zymogen FXI and the active protease homolog, PKa [16,17], structures for FXIa, PK, and full-length FIX or its intermediates are absent. Structural models of FXIa relied on homology modeling after PKa. In the crystal structure of PKa, the CD is rotated 180° relative to its apple domain platform when compared directly with the FXI crystal structure. Extrapolating this 180° spin to FXIa generated a model where a macrorotation of the FXI-CD is proposed to take place following zymogen activation [25]. We solved the cryo-EM structures of the complexes FXIa:FIX and FXIa:FIXaβ resolving this ambiguity. We demonstrated that no significant rotational transition occurs upon activation, at least relevant to FIX activation. Instead, we visualized an elegant, rigid-body pivoted tipping motion of the FXIa-CD toward the adjacent FXIa-A4. This tilting action spatially unmasks more of the A3 topology for unhindered FIX engagement while positioning the FXIa-CD into a functionally relevant conformation for processing the FIX-AP. This active conformation is stabilized structurally by the newly cleaved C-terminus of the FXIa HC, which acts as an internal stabilization hub mediating extensive contacts with the A1-A2 and A3-A4 linkers reinforced by direct CD-to-A3 and CD-to-A4 anchoring contacts. To our knowledge, the new structures provide the first complete view of full-length FIX and FIXaβ, capturing the first step of proteolysis at Arg145 (for FIX) and an intriguing retethering of FIX-Arg145 (for FIXaβ) within the FXIa-CD S1 pocket following the secondary cleavage at Arg180. This retethering is likely driven by stabilization of the new FIX-Arg145 carboxylate moiety by the basic FXIa-Lys554 side chain. FXIa-Lys554 is positioned directly at the rim framing the S1 pocket and oxyanion hole and acts as an electrostatic anchor, in direct contrast to the equivalent (E192)c in thrombin. Crucially, this work provides a direct structural demonstration of the FIX-Gla domain engaging the FXIa-A3 exosite, validating decades of biochemical studies.

Although our data capture the initial docking of FIX, defining the limits of these structures allows us to explore broader physiological questions. First, it is highly plausible that the CD in free FXIa, and to a lesser extent in zymogen FXI, samples a dynamic ensemble of conformations in solution. This structural plasticity would allow diverse substrates or endogenous inhibitors to differentially engage the FXI/FXIa apple domain platform. This observation raises compelling future questions regarding its homologous counterpart: how is the CD positioned in zymogen PK vs active PKa, and whether these distinct states rely, similarly, on highly dynamic structural ensembles allowing various targets’ engagement? Furthermore, 2 specific physiological mechanisms remain unanswered. The first relates to the precise spatial configuration required for the secondary cleavage at FIX-Arg180. While not directly visualized in a transition state, our data shed light on the relative flexibility of the FIX EGF and CDs compared with the rigidly tethered Gla domain. We hypothesize that this inherent flexibility provides a sufficient stochastic range of motion to allow the FIX-CD to hover dynamically above the FXIa active site, elegantly presenting Arg180 for processing without requiring the substrate to fully unbind and rebind to the FXIa platform. Second, the physiological necessity of the unique dimeric architecture of FXI *in vivo* remains to be fully explained. Our structural findings show that the FXIa dimer functions as a pair of autonomous proteolytic centers when both subunits are engaged in FIX activation. The rigid Apple platform shows no more than molecular breathing-level movement across all heterogeneity analyses and hence appears to serve purely as a localized scaffold rather than a mediator of allosteric catalytic cooperativity. This structural autonomy is supported by *in vitro* biochemical data demonstrating that monomeric FXIa variants activate FIX at rates indistinguishable from the wild-type dimer [41]. Consequently, the evolutionary requirement for the dimer *in vivo* likely relates to the simultaneous, asymmetric engagement of FXI subunits with disparate substrates or binding partners as receptors at the activated platelet surface, which has been proposed previously [53]. Whether this apparent independence of FXIa subunits, or the rigidity of the apple platform itself, is preserved when the 2 subunits engage distinct substrates or partners remains an open question. Resolving the architecture of these complexes in their native membrane-bound microenvironments, and the new interface residues defined here, represents the next frontier of our ongoing structural investigations.

Ultimately, the new structures establish a framework for ongoing biochemical validation of interface residues and future investigations into the multicomponent assemblies of the contact activation pathway, including factor XII and PK. Crucially, the structures provide a useful framework for future structure-guided development of compounds targeting the FIX substrate recognition mechanism by FXIa. Current clinical trials feature several active-site and antibody-based FXI inhibitors. Defining the FXIa:FIX interface at high resolution opens the door for the development of a new class of selective small-molecule allosteric inhibitors or peptidomimetics. By targeting these newly defined, noncatalytic pockets, it could be feasible to decouple FXIa from its primary substrate without risking active-site cross-reactivity with homologous serine proteases. Supported by preclinical precedents in murine thrombosis models [54], this structure-guided paradigm will offer an additional molecular strategy to elegantly isolate pathologic thrombosis from baseline hemostasis, driving the development and diversification of a safer antithrombotics portfolio.

## Supplementary Material

Supplementary Material

Supplementary Video S1

Supplementary Video S2

Supplementary Video S3

Supplementary Video S4

Supplementary Video S5

The online version contains supplementary material available at https://doi.org/10.1016/j.jtha.2026.08.015.

## Figures and Tables

**FIGURE 1:**
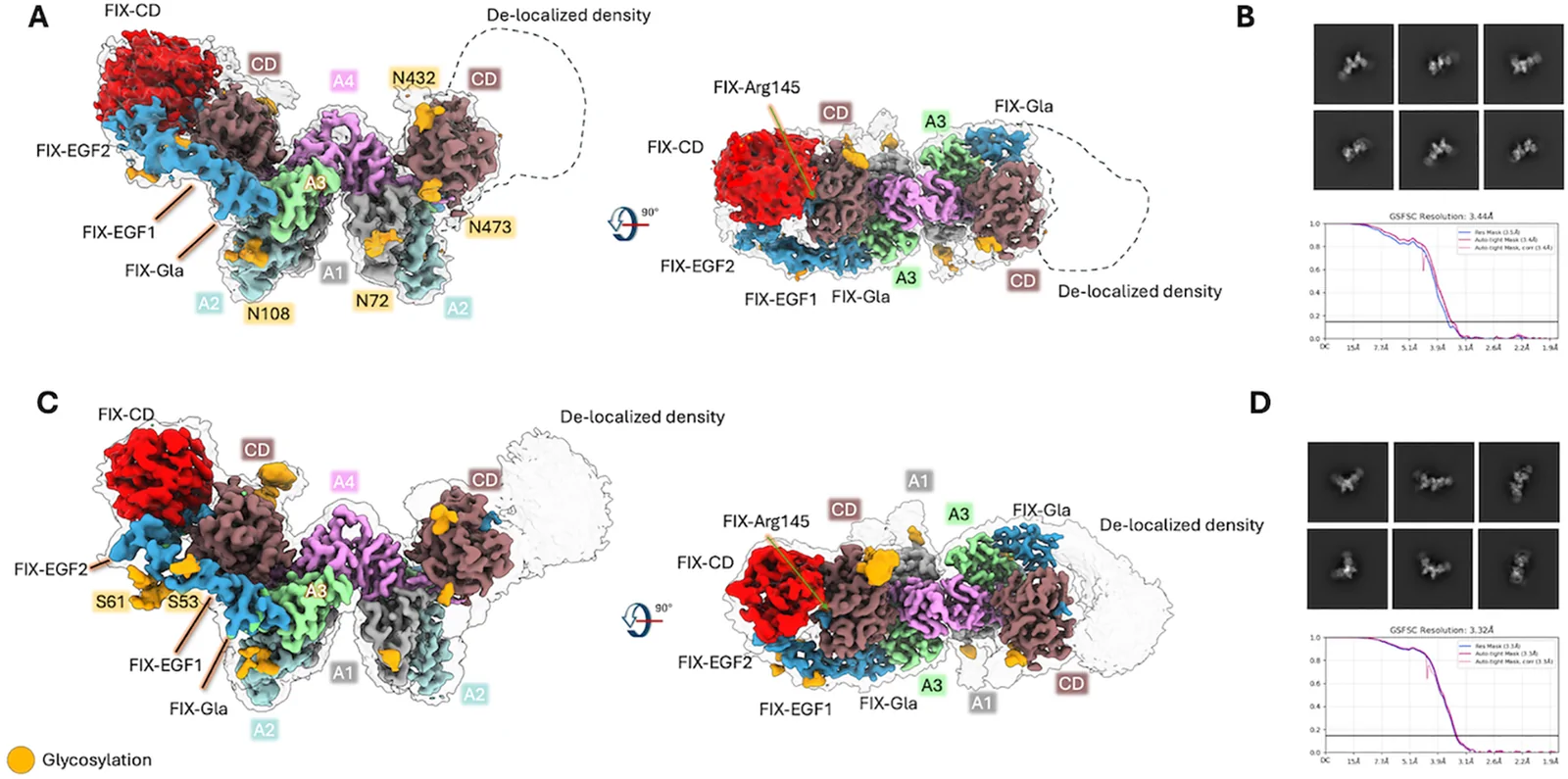
Architecture of the human FXIa:FIX and FXIa:FIXaβ complexes. (A) Cryo-electron microscopy (cryo-EM) density maps of the FXIa:FIX zymogen complex shown in 2 orthogonal views. The global consensus map (EMD-76284) is rendered as a transparent white surface with composite map (EMD-76288) showing local reconstructions of different regions superposed. (B) Representative reference-free 2D class averages and GSFSC, gold-standard Fourier shell correlation. (C, D) Corresponding cryo-EM density maps EMD-74884 (consensus), EMD-74894 (composite), and reference-free 2D class averages and GSFSC for the FXIa:FIXaβ complex. N- and O-linked glycosylation sites are highlighted in orange and labeled for FXIa in panel (A) and for FIX in panel (C). In the top views of both complexes (A and C), the rigid FIX-Gla domain is clearly resolved docked onto both subunits of the FXIa homodimer. As detailed in the text, the FIX EGF1, EGF2, and catalytic domains (CD) exhibit a range of motion due to flexible linkers, resulting in the FIX-CD hovering over the FXIa-CD. FIX-Arg145 is highlighted in the top view maps. At the displayed contour levels, this diffused hovering density corresponding to FIX-CD persisted in the consensus map of the FXIa:FIXaβ complex but was not visible in the FXIa:FIX complex (shown as dotted line). Diffuse density corresponding to the mobile FIX regions on the second FXIa subunit is further detailed in Supplementary Figure S2.

**FIGURE 2:**
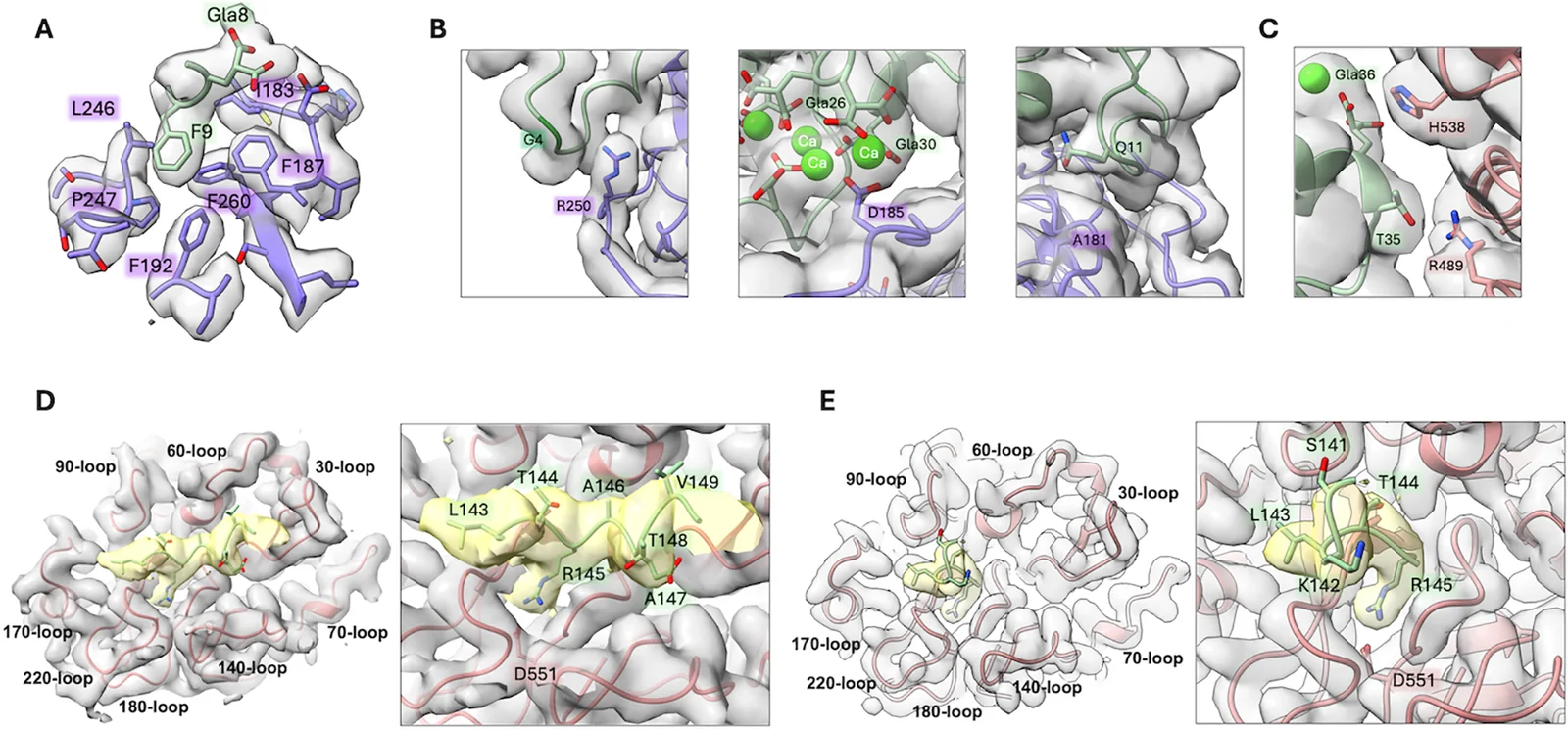
The FXIa:FIX interface. (A) Cryo-electron microscopy (cryo-EM) map and corresponding atomic model detailing the primary docking interface, highlighting FIX-Phe9 (green) of the Ω−loop sequestered within the shallow hydrophobic pocket of the FXIa-A3 domain (purple). (B) Structural details of the auxiliary clamping contacts at the N- and C-termini of the FIX Ω−loop (green) with the FXIa-A3 domain (purple) as detailed in the text. (C) Visualization of the secondary contact interface between the FIX-Gla domain (green) and the FXIa catalytic domain (FXIa-CD, coral). (D) Cryo-EM map and model of the FXIa:FIX zymogen complex with the FXIa-CD (coral) displayed in the standard Bode orientation (the 170-loop on the left, and the S1 specificity pocket, composed of the 220- and 180-loops, oriented downward). Cryo-EM density corresponding to the FIX-AP (green sticks) is highlighted with a transparent yellow surface. The right panel provides a magnified view of the FIX-AP natively seated within the active site cleft. (E) Corresponding cryo-EM map and model for the activated FXIa:FIXaβ complex, detailing the postcleavage retethering of the substrate P1 residue.

**FIGURE 3:**
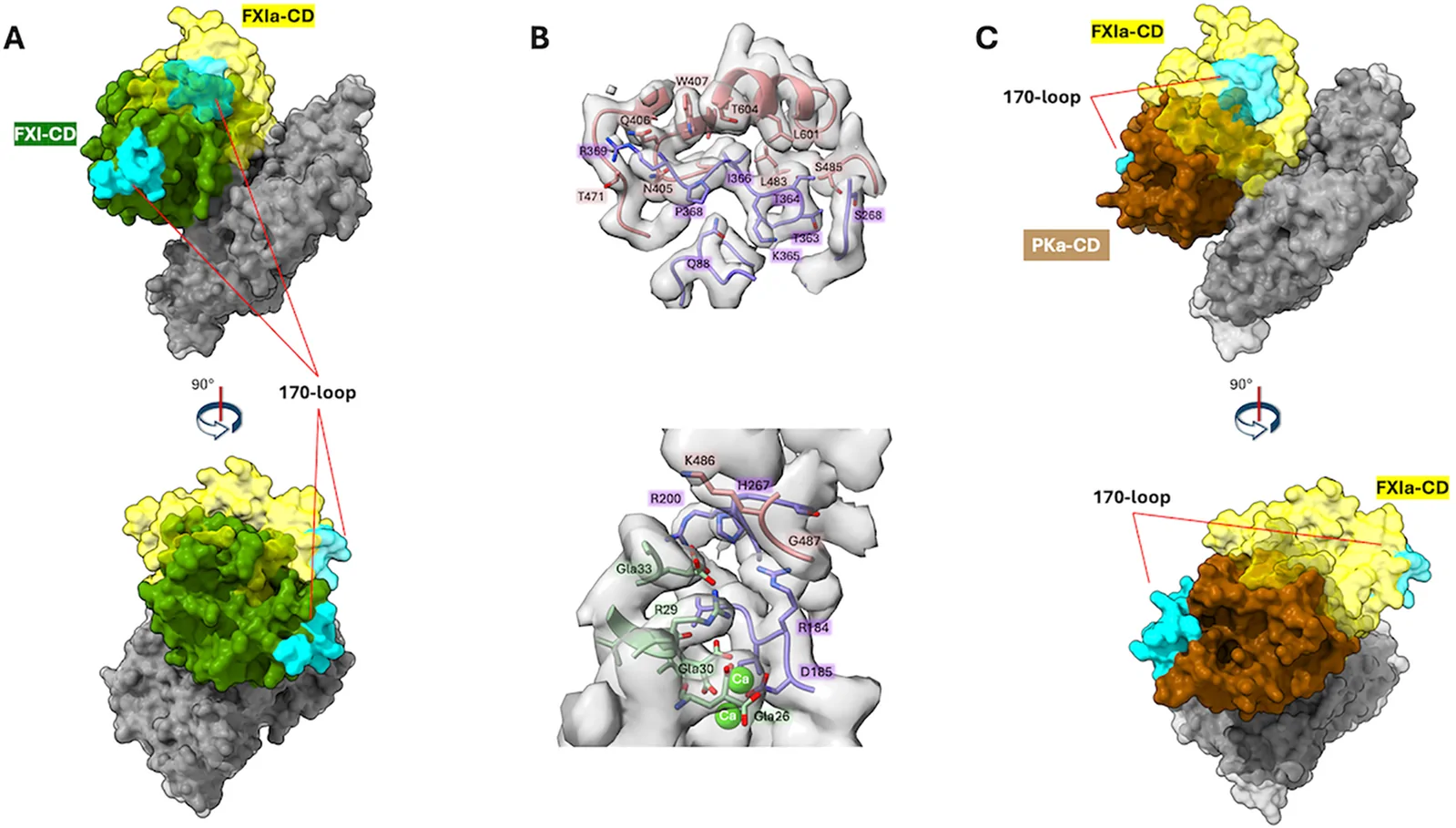
Factor XI activation-associated functional conformational change and divergence from plasma kallikrein. (A) Activation of FXI induces a rigid-body pivoted tipping of the catalytic domain (CD) toward the A4 domain, without significant macrorotation. Superposition of a single subunit of the zymogen FXI heavy chain (Protein Data Bank [PDB] 6I58; solid gray) with the active FXIa heavy chain (transparent gray) highlights a significant upward displacement of the active CD (transparent yellow) relative to the zymogen CD (green). The 170-loop (cyan) is highlighted as a structural landmark to trace this upward trajectory. (B) Stabilization of the FXIa active conformation in the complex. *Top:* The FXIa-CD (coral) is anchored by a network of interactions involving the newly cleaved FXIa-HC C-terminus, the A1-A2 linker, and the A3-A4 linker. *Bottom:* view of the structural coordination mediated by biochemically studied and clinically relevant residues Arg184 and Asp185 in active FXIa. (C) Structural divergence between FXIa and plasma kallikrein (PKa). Alignment of the free PKa (PDB 6O1G [17]; solid gray) with the active FXIa subunit (transparent gray) by their respective heavy chains demonstrates that the FXIa-CD (transparent yellow) adopts a fundamentally distinct functional orientation compared with the 180° -rotated PKa-CD model (brown). The 170-loop (cyan) is highlighted as in (A).

**TABLE 1:** Cryo-EM data collection parameters, refinement, and validation statistics for FXIa:FIX and FXIa:FIXaβ complexes.

Accession codes	FXIa-FIX	FXIa-FIXaβ
	PDB 12BN EMD-76288	PDB 9ZVX EMD-74894
**Data Collection and Processing**		
Nominal magnification	150 000×	
Voltage (kV)	200	
Electron exposure (e^−^/Å^2^)	55	
Defocus range (μm)	−1 to −2.4 μm	
Pixel size (Å)	0.928	
Number of images	11 188	11 397
Final particle images	87 309	224 103
Symmetry imposed	C1	
Resolution (masked, Fourier shell correlation threshold 0.143)	3.4	3.3
**Model Composition**		
Refinement program	Phenix (real space)	
Number of protein atoms (non-H)	13 151	7 841
Protein residues	1644	985
Ligand molecules	NAG (8) Ca (16)	NAG (4) Ca (8)
**R.m.s.d. Deviations**		
Bond lengths (Å)	0.003 (0)	0.004 (0)
Bond angles (°)	0.613 (0)	0.770 (0)
**Validation**		
All-atom clash score	7.73	9.32
Poor Rotamers (%)	0.0	0.0
Ramachandran disallowed (%)	0.0	0.0
Ramachandran allowed (%)	4.77	6.33
Ramachandran favored (%)	95.23	93.67
MolProbity score	1.75	1.91

**TABLE 2:** Hierarchical interaction network stabilizing the active FXIa catalytic domain.

Domain Involved	Specific Connection
**I. Direct Domain Anchoring**	
CD and A4	Glu567 → Phe277 and Ser275
	Val568 →Ser275
CD and A3	Ser485 →Ser268
	Lys486 →His267
	Gly487 →Arg184
**II. Stabilization Hub (CD to AP)**	
CD and AP	Asn405 →Pro368 and Arg369
	Gln406, Asn405, and Thr471 →Arg369
	Tyr474 →Met358
	Arg479 →Glu361 and Met358
	Pro480 →Asp359
	Ser485 →Thr364, Thr363 (AP), and Ser268 (A3-A4 linker)
	Ile601, Ile600, Leu483, Thr604, and Trp407 →Ile366
**III. Secondary AP tethering to HC**	
AP and A1-A2 Linker	Lys357 →Ser86
	Met358 →Ser86
	Lys365 →Gln88
	Pro368 →Gln88
AP and A3-A4 Linker	Glu361 and Asn360 →Val271
	Thr363 →Ser268 and Val271
	Thr364 →Ser268

The table details the specific noncovalent interaction network that locks the activated FXIa catalytic domain (CD) into its functional orientation. The architecture is organized into 3 hierarchical levels: (I) direct structural anchoring of the CD to the underlying Apple 3 (A3) and Apple 4 (A4) domains; (II) a central stabilization hub formed between the CD and the new C-terminus of the FXIa-HC (the activation peptide region-AP); and (III) secondary tethering of the AP to the A1-A2 and A3-A4 linkers. Specific residue-to-residue contacts are listed, with arrows denoting directional polar interactions (eg, hydrogen bonds or salt bridges) or primary points of van der Waals contact.
