# Supplementary material for: Structural insights into the exosite-mediated activation of factor IX by factor XIa using cryogenic electron microscopy
Source: J Thromb Haemost. Author manuscript; Available in PMC 2026 Oct 14. (PMC13663045; doi:10.1016/j.jtha.2026.08.015)
Supplement: Supplementary file 1 [file NIHMS2213686-supplement-Supplementary_Material.docx]

**Supplementary Information**

**Structural insights into the exosite-mediated activation of Factor IX by Factor XIa using cryo-EM**

**Bassem M. Mohammed^1#^, Samantha Deavila^1^, Tristan Friet^1^, Isabella Dattilio^1^**

^1^Edward A. Doisy Department of Biochemistry and Molecular Biology, Saint Louis University School of Medicine, St. Louis, MO 63104 USA

**Supplementary Figure S1.** SDS-PAGE analysis of purified recombinant and plasma-derived proteins. Samples were resolved under non-reducing (A) and reducing conditions (B) to assess integrity and confirm purity. (C) SDS-PAGE analysis of plasma-derived FIXaβ under non-reducing (NR), and reducing (R) conditions showing FIX heavy chain (HC) and light chain (LC). Molecular weight markers in kiloDaltons (kDa) are indicated on the left of all panels.

**Supplementary Figure S2.** Cryo-EM data processing workflows performed in CryoSPARC for (A) the FXIa:FIX complex, and (B) FXIa:FIXaβ complex. In **(A)**, the final reconstruction EMD-76284 (gray) features an orange asterisk highlighting extra density atop the FXIa-CD:FIX-CD interface shown at low isosurface, which is likely attributed to post-translational glycosylation within the FIX activation peptide (unmodeled in the final coordinate). The red asterisk denotes the highly delocalized density corresponding to FIX on the second FXIa subunit (outlined by the dotted line in Figure 1A consensus map EMD-76284). This map was subjected to focused masked refinement to generate the final composite map EMD-76288 and atomic model PDB 12BN for the FXIa:FIX complex. In **(B)**, Class 3 consensus map EMD-74884 underwent analogous masked refinement as in (A) to yield the final high-resolution composite map EMD-74894 and model PDB 9ZVX for the FXIa:FIXaβ complex. Classes 1, 2, and 4 were independently processed and deposited to structurally capture the dynamic hovering motion of the FIX-CD above the FXIa-CD.

**Supplementary Figure S3.** Cryo-EM density maps and corresponding atomic models for **(A)** FXIa:FIX (left panel, EMD-76288 and PDB 12BN) and FXIa:FIXaβ (right panel, EMD-74894 and PDB 9ZVX). **(B)** Local resolution estimates mapped onto the surface representation of the FXIa:FIXaβ complex rigid core (EMD-74584). This focused reconstruction was generated using a mask covering the FXIa homodimer and the FIX-Gla domain on each FXIa subunit. The resulting high-resolution coordinate (PDB 9ZQY) was utilized as the structural template to model the FXIa subunit within the full-length macromolecular complexes. **(C)** FIXaβ catalytic domain (CD) highlighting the insertion of the newly formed N-terminus, Val181 (green), and its interaction with Asp364 (blue). The backbone density for both residues is rendered transparent to clearly display the residues which are presented as sticks. Chymotrypsin numbering is indicated in brackets followed by lowercase “c”. **(D)** Local resolution estimates mapped onto the surface representation of the FXIa:FIX (left panel, EMD-76288) and FXIa:FIXaβ (right panel, EMD-74894) composite maps.

**Supplementary Figure S4.** Conformational dynamics captured for the activated FXIa:FIXaβ complex. (A) Superposed space-filling models of FXIa:FIXaβ Classes 1, 2, 3, and 4 displayed in three orthogonal views. The superposition illustrates the broad conformational range and continuous hovering motion of the FIX domains, corresponding to the dynamics captured in Supplementary Video 3. **(B)** Corresponding cartoon representations of the same superposed classes in the same orientation as (A middle panel), detailing the underlying flexibility of the EGF1, EGF2, and FIX-CD about the inter-domain linkers.

**Supplementary Table S1.** Cryo-EM data collection, refinement, and validation statistics for the FXIa:FIXaβ complex rigid core and classes 1,2 and 4. The complex rigid core was modeled *ab-initio*, whereas Classes 1, 2, and 4 were built using a hybrid approach. For these dynamic classes, the refined complex core served as the initial template, followed by rigid-body fitting and backbone tracing to assign the remaining flexible domains of FIXaβ.

|  | FXIa:FIX-Gla rigid core | Class 1 | Class 2 | Class 4 |
| --- | --- | --- | --- | --- |
| Accession codes | PDB 9ZQY  EMD-74584 | PDB 9ZTK  EMD-74856 | PDB 9ZUB  EMD-74802 | PDB 9ZUN  EMD-74822 |
| **Data collection and processing** | | | | |
| Nominal Magnification | 150,000x | | | |
| Voltage (kV) | 200 | | | |
| Electron exposure (e-/Å^2^) | 55 | | | |
| Defocus range ($\mu$m) | −1 to −2.4 μm | | | |
| Pixel size (Å) | 0.928 | | | |
| Number of images | 11,397 | | | |
| Final particle images | 682,170 | 103,693 | 78,076 | 121,893 |
| Symmetry imposed | C1 | C1 | C1 | C1 |
| Resolution (masked, FSC threshold 0.143) | 3.09 | 3.70 | 3.65 | 3.55 |
| **Model Composition** | | | | |
| Refinement program | Phenix (real space) | | | |
| Number of protein atoms (non-H) | 10624 | 7882 | 7860 | 7878 |
| Protein residues | 1316 | 988 | 986 | 988 |
| Ligand molecules | Ca (16)  NAG (8) | Ca (7)  NAG (4) | Ca (8)  NAG (4) | Ca (8)  NAG (4) |
| **R.m.s.d. deviations** | | | | |
| Bond lengths (Å) | 0.004 (0) | 0.005 (0) | 0.004 (0) | 0.004 (1) |
| Bond angles (°) | 0.846 (12) | 0.857 (8) | 0.787 (2) | 0.753 (4) |
| **Validation** | | | | |
| All-atom clash score | 9.52 | 13.4 | 11.3 | 9.2 |
| Poor Rotamers (%) | 0.35 | 0.35 | 0.23 | 0.23 |
| Ramachandran disallowed (%) | 0.0 | 0.0 | 0.0 | 0.0 |
| Ramachandran allowed (%) | 5.07 | 4.73 | 5.37 | 4.1 |
| Ramachandran favored (%) | 94.93 | 95.27 | 94.63 | 95.90 |
| MolProbity score | 1.85 | 1.97 | 1.94 | 1.77 |

**Supplementary Table S2.** Comprehensive inter-domain contact network for the FXIa:FIX generated using PDBsum^45^. Within the table, light blue highlight indicates hydrogen bonding interactions and dark blue highlight denotes the presence of salt bridges. All other non-bonded contacts (van der Waals interactions) remain unhighlighted. Abbreviations: CGU is the PDB code for a Gamma-carboxyglutamate (Gla) residue.

| **No.** | **FXIa-LC** | **FIX** | **Distance (Å)** | **No.** | **FXI-HC** | **FIX-Gla** | **Distance (Å)** |
| --- | --- | --- | --- | --- | --- | --- | --- |
| 1 | Arg489 (NH1) | Thr35 (CB) | 3.59 | 1 | Arg250 (NH1) | Gly4 (O) | 3.12 |
| 2 | Arg489 (NH1) | Thr35 (OG1) | 2.84 | 2 | Ser248 (O) | Leu6 (CD2) | 3.47 |
| 3 | His538 (CE1) | CGU36 (OE22) | 3.01 | 3 | Asp185 (OD2) | CGU8 (OE11) | 3.85 |
| 4 | His538 (NE2) | CGU36 (OE22) | 3.25 | 4 | Phe187 (CE2) | CGU8 (OE21) | 3.56 |
| 5 | Tyr416 (OH) | Ser136 (O) | 3.55 | 5 | Asp185 (OD2) | CGU8 (OE22) | 3.66 |
| 6 | Tyr416 (OH) | Val137 (CA) | 3.57 | 6 | Leu246 (CD2) | Phe9 (O) | 3.72 |
| 7 | Tyr416 (OH) | Val137 (C) | 3.63 | 7 | Phe260 (CE2) | Phe9 (CG) | 3.87 |
| 8 | Tyr416 (CE2) | Ser138 (N) | 3.82 | 8 | Phe260 (CZ) | Phe9 (CG) | 3.64 |
| 9 | Tyr416 (CZ) | Ser138 (N) | 3.7 | 9 | Phe260 (CE2) | Phe9 (CD1) | 3.75 |
| 10 | Tyr416 (OH) | Ser138 (N) | 2.86 | 10 | Phe260 (CZ) | Phe9 (CD1) | 3.63 |
| 11 | Tyr416 (OH) | Ser138 (CA) | 3.66 | 11 | Pro247 (CG) | Phe9 (CD2) | 3.78 |
| 12 | Tyr416 (CE2) | Ser138 (O) | 3.84 | 12 | Phe260 (CZ) | Phe9 (CD2) | 3.84 |
| 13 | Tyr416 (CZ) | Ser138 (O) | 3.89 | 13 | Phe187 (CE1) | Phe9 (CE1) | 3.77 |
| 14 | Tyr416 (OH) | Ser138 (CB) | 3.49 | 14 | Phe187 (CZ) | Phe9 (CE1) | 3.45 |
| 15 | Tyr416 (OH) | Ser138 (OG) | 3.78 | 15 | Phe260 (CZ) | Phe9 (CE1) | 3.81 |
| 16 | Glu579 (O) | Lys142 (O) | 3.68 | 16 | Pro247 (CG) | Phe9 (CE2) | 3.39 |
| 17 | Glu579 (O) | Leu143 (CA) | 3.9 | 17 | Ala181 (CB) | Gln11 (CG) | 3.51 |
| 18 | Trp577 (CA) | Leu143 (O) | 3.78 | 18 | Asp185 (OD2) | CGU30 (CD2) | 3.5 |
| 19 | Trp577 (CB) | Leu143 (O) | 3.21 | 19 | Asp185 (CG) | CGU30 (OE11) | 3.72 |
| 20 | Gly578 (N) | Leu143 (O) | 3.5 | 20 | Asp185 (OD1) | CGU30 (OE11) | 3.07 |
| 21 | Glu579 (N) | Leu143 (O) | 3.54 | 21 | Asp185 (OD2) | CGU30 (OE11) | 3.89 |
| 22 | His534 (NE2) | Leu143 (CD1) | 3.35 | 22 | Asp185 (OD2) | CGU30 (OE21) | 3.73 |
| 23 | Glu579 (CA) | Leu143 (CD2) | 3.52 | 23 | Asp185 (CG) | CGU30 (OE22) | 3.69 |
| 24 | Glu579 (C) | Leu143 (CD2) | 3.73 | 24 | Asp185 (OD1) | CGU30 (OE22) | 3.59 |
| 25 | Glu579 (O) | Leu143 (CD2) | 3.62 | 25 | Asp185 (OD2) | CGU30 (OE22) | 3 |
| 26 | Glu579 (CG) | Leu143 (CD2) | 3.73 | 26 | His267 (CD2) | CGU33 (CD1) | 3.49 |
| 27 | Ala457 (CB) | Thr144 (CG2) | 3.56 | 27 | His267 (CG) | CGU33 (OE11) | 3.64 |
| 28 | His413 (CD2) | Arg145 (N) | 3.66 | 28 | His267 (CD2) | CGU33 (OE11) | 2.98 |
| 29 | His413 (NE2) | Arg145 (N) | 3.31 | 29 | His267 (NE2) | CGU33 (OE11) | 3.34 |
| 30 | Ser576 (O) | Arg145 (N) | 3.69 | 30 | His267 (CD2) | CGU33 (OE12) | 3.75 |
| 31 | His413 (NE2) | Arg145 (CA) | 3.47 |  |  |  |  |
| 32 | His413 (NE2) | Arg145 (C) | 3.41 |  |  |  |  |
| 33 | Ala557 (CB) | Arg145 (C) | 3.73 |  |  |  |  |
| 34 | Lys554 (C) | Arg145 (O) | 3.81 |  |  |  |  |
| 35 | Lys554 (O) | Arg145 (O) | 3.87 |  |  |  |  |
| 36 | Ala557 (CB) | Arg145 (O) | 3.47 |  |  |  |  |
| 37 | His413 (NE2) | Arg145 (CB) | 3.27 |  |  |  |  |
| 38 | Ala557 (CA) | Arg145 (CB) | 3.69 |  |  |  |  |
| 39 | Ala557 (CB) | Arg145 (CB) | 3.82 |  |  |  |  |
| 40 | Ser576 (O) | Arg145 (CB) | 3.89 |  |  |  |  |
| 41 | Thr575 (CG2) | Arg145 (CG) | 3.57 |  |  |  |  |
| 42 | Ser576 (C) | Arg145 (CG) | 3.79 |  |  |  |  |
| 43 | Ser576 (O) | Arg145 (CG) | 3.56 |  |  |  |  |
| 44 | Trp577 (N) | Arg145 (CG) | 3.81 |  |  |  |  |
| 45 | Trp577 (CA) | Arg145 (CG) | 3.56 |  |  |  |  |
| 46 | Trp577 (C) | Arg145 (NE) | 3.55 |  |  |  |  |
| 47 | Gly578 (N) | Arg145 (NE) | 3.33 |  |  |  |  |
| 48 | Gly578 (CA) | Arg145 (NE) | 3.79 |  |  |  |  |
| 49 | Ala552 (C) | Arg145 (CZ) | 3.7 |  |  |  |  |
| 50 | Ala552 (O) | Arg145 (CZ) | 3.09 |  |  |  |  |
| 51 | Gly578 (N) | Arg145 (CZ) | 3.58 |  |  |  |  |
| 52 | Gly578 (CA) | Arg145 (CZ) | 3.56 |  |  |  |  |
| 53 | Ala552 (C) | Arg145 (NH1) | 3.33 |  |  |  |  |
| 54 | Ala552 (O) | Arg145 (NH1) | 2.76 |  |  |  |  |
| 55 | Cys553 (N) | Arg145 (NH1) | 3.53 |  |  |  |  |
| 56 | Cys553 (CA) | Arg145 (NH1) | 3.2 |  |  |  |  |
| 57 | Lys554 (N) | Arg145 (NH1) | 3.62 |  |  |  |  |
| 58 | Asp551 (OD2) | Arg145 (NH2) | 2.81 |  |  |  |  |
| 59 | Ala552 (C) | Arg145 (NH2) | 3.84 |  |  |  |  |
| 60 | Ala552 (O) | Arg145 (NH2) | 2.98 |  |  |  |  |
| 61 | Gly578 (N) | Arg145 (NH2) | 3.6 |  |  |  |  |
| 62 | Gly578 (CA) | Arg145 (NH2) | 3.1 |  |  |  |  |
| 63 | Cys581 (O) | Arg145 (NH2) | 3.84 |  |  |  |  |
| 64 | His413 (CD2) | Ala146 (N) | 3.17 |  |  |  |  |
| 65 | His413 (NE2) | Ala146 (N) | 3.15 |  |  |  |  |
| 66 | His413 (CD2) | Ala146 (CA) | 3.8 |  |  |  |  |
| 67 | Lys554 (NZ) | Ala146 (O) | 3.09 |  |  |  |  |
| 68 | His413 (O) | Ala146 (CB) | 3.54 |  |  |  |  |
| 69 | His413 (CD2) | Ala146 (CB) | 3.61 |  |  |  |  |
| 70 | Cys414 (CA) | Ala146 (CB) | 3.78 |  |  |  |  |
| 71 | Lys554 (O) | Glu147 (CA) | 3.58 |  |  |  |  |
| 72 | Leu397 (O) | Glu147 (CB) | 3.87 |  |  |  |  |
| 73 | Lys554 (O) | Glu147 (CB) | 3.4 |  |  |  |  |
| 74 | Lys554 (O) | Glu147 (CG) | 3.51 |  |  |  |  |
| 75 | Arg395 (CZ) | Glu147 (OE1) | 3.88 |  |  |  |  |
| 76 | Arg395 (NH1) | Glu147 (OE1) | 2.88 |  |  |  |  |
| 77 | His396 (O) | Glu147 (OE1) | 3.84 |  |  |  |  |
| 78 | Tyr503 (OH) | Glu147 (OE2) | 3.33 |  |  |  |  |
| 79 | Lys554 (NZ) | Thr148 (N) | 3.52 |  |  |  |  |
| 80 | Arg395 (CZ) | Thr148 (O) | 3.81 |  |  |  |  |
| 81 | Arg395 (NH1) | Thr148 (O) | 3.67 |  |  |  |  |
| 82 | Lys554 (CE) | Thr148 (OG1) | 3.63 |  |  |  |  |
| 83 | Lys554 (NZ) | Thr148 (OG1) | 3.5 |  |  |  |  |
| 84 | Arg395 (NH2) | Val149 (CA) | 3.77 |  |  |  |  |
| 85 | Arg395 (NH2) | Val149 (C) | 3.67 |  |  |  |  |
| 86 | Arg395 (CZ) | Val149 (O) | 3.69 |  |  |  |  |
| 87 | Arg395 (NH2) | Val149 (O) | 2.95 |  |  |  |  |
| 88 | Pro392 (O) | Asp186 (CG) | 3.79 |  |  |  |  |
| 89 | Pro392 (CB) | Asp186 (CG) | 3.83 |  |  |  |  |
| 90 | Thr393 (OG1) | Asp186 (CG) | 3.58 |  |  |  |  |
| 91 | Pro392 (CA) | Asp186 (OD1) | 3.3 |  |  |  |  |
| 92 | Pro392 (C) | Asp186 (OD1) | 3.33 |  |  |  |  |
| 93 | Pro392 (O) | Asp186 (OD1) | 3.2 |  |  |  |  |
| 94 | Pro392 (CB) | Asp186 (OD1) | 3.24 |  |  |  |  |
| 95 | Thr393 (OG1) | Asp186 (OD1) | 3.87 |  |  |  |  |
| 96 | Pro392 (C) | Asp186 (OD2) | 3.65 |  |  |  |  |
| 97 | Pro392 (O) | Asp186 (OD2) | 3.69 |  |  |  |  |
| 98 | Pro392 (CB) | Asp186 (OD2) | 3.78 |  |  |  |  |
| 99 | Thr393 (CB) | Asp186 (OD2) | 3.41 |  |  |  |  |
| 100 | Thr393 (OG1) | Asp186 (OD2) | 2.82 |  |  |  |  |
| 101 | Arg507 (NH1) | Glu355 (CB) | 3.23 |  |  |  |  |
| 102 | Arg507 (NH1) | Glu355 (CG) | 3.75 |  |  |  |  |
| 103 | Arg507 (NH2) | Arg358 (CG) | 3.86 |  |  |  |  |
| 104 | Arg507 (CZ) | Arg358 (CD) | 3.54 |  |  |  |  |
| 105 | Arg507 (NH1) | Arg358 (CD) | 3.21 |  |  |  |  |
| 106 | Arg507 (NH2) | Arg358 (CD) | 3.54 |  |  |  |  |
| 107 | Arg507 (CZ) | Arg358 (NE) | 3.88 |  |  |  |  |
| 108 | Arg507 (NH2) | Arg358 (NE) | 3.84 |  |  |  |  |

**Supplementary Table S3.** Comprehensive inter-domain contact network for the FXIa:FIXaβ generated using PDBsum^45^. Within the table, light blue highlight indicates hydrogen bonding interactions and dark blue highlight denotes the presence of salt bridges. All other non-bonded contacts (van der Waals interactions) remain unhighlighted. CGU is the PDB code for a Gamma-carboxyglutamate (Gla) residue.

| **No.** | **FXIa-HC** | **FIXaβ-LC** | **Distance (Å)** | **No.** | **FXIa - CD** | **FIXaβ-LC** | **Distance (Å)** |
| --- | --- | --- | --- | --- | --- | --- | --- |
| 1 | Ala181 (CB) | Gln11 (CB) | 3.62 | 1 | His413 (CG) | Thr144 (OG1) | 3.45 |
| 2 | Ile183 (CD1) | CGU8 (O) | 3.48 | 2 | His413 (CD2) | Thr144 (OG1) | 3.02 |
| 3 | Ile183 (CD1) | Phe9 (CA) | 3.57 | 3 | His413 (NE2) | Thr144 (OG1) | 3.41 |
| 4 | Ile183 (CD1) | Phe9 (C) | 3.31 | 4 | His413 (NE2) | Arg145 (N) | 3.83 |
| 5 | Ile183 (CD1) | Phe9 (O) | 3.18 | 5 | His413 (NE2) | Arg145 (CB) | 3.53 |
| 6 | Asp185 (CG) | CGU30 (OE11) | 3.43 | 6 | Tyr416 (O) | Ser136 (CB) | 3.36 |
| 7 | Asp185 (CG) | CGU30 (OE22) | 3.77 | 7 | Tyr416 (O) | Ser136 (OG) | 3.82 |
| 8 | Asp185 (OD1) | CGU30 (OE11) | 3.09 | 8 | Tyr416 (CD2) | Ser136 (CB) | 3.88 |
| 9 | Asp185 (OD2) | CGU30 (CD2) | 3.21 | 9 | Tyr416 (CD2) | Thr140 (OG1) | 3.69 |
| 10 | Asp185 (OD2) | CGU30 (OE11) | 3.27 | 10 | Tyr416 (CE2) | Val135 (O) | 3.89 |
| 11 | Asp185 (OD2) | CGU30 (OE21) | 3.53 | 11 | Tyr416 (CZ) | Arg134 (CD) | 3.73 |
| 12 | Asp185 (OD2) | CGU30 (OE22) | 2.74 | 12 | Tyr416 (OH) | Arg134 (CD) | 3.12 |
| 13 | Phe187 (CE2) | Phe9 (CD1) | 3.79 | 13 | Tyr416 (OH) | Arg134 (NE) | 3.16 |
| 14 | Phe187 (CE2) | Phe9 (CE1) | 3.25 | 14 | Tyr416 (OH) | Arg134 (CZ) | 3.03 |
| 15 | Phe187 (CZ) | CGU8 (O) | 3.47 | 15 | Tyr416 (OH) | Arg134 (NH1) | 2.82 |
| 16 | Phe192 (CE1) | Phe9 (CZ) | 3.64 | 16 | Tyr416 (OH) | Arg134 (NH2) | 3.88 |
| 17 | Phe192 (CZ) | Phe9 (CZ) | 3.5 | 17 | Gly417 (CA) | Ser136 (CB) | 3.59 |
| 18 | Leu246 (CD1) | Phe9 (O) | 3.89 | 18 | Gly417 (CA) | Ser136 (OG) | 3.75 |
| 19 | Pro247 (O) | Phe9 (CE2) | 3.9 | 19 | Glu419 (CD) | Pro129 (O) | 3.85 |
| 20 | Pro247 (CG) | Phe9 (CE2) | 3.37 | 20 | Glu419 (CD) | Pro129 (CB) | 3.89 |
| 21 | Pro247 (CD) | Phe9 (CE2) | 3.8 | 21 | Glu419 (CD) | Phe130 (CE2) | 3.58 |
| 22 | Ser248 (O) | Leu6 (CD2) | 3.33 | 22 | Glu419 (CD) | Phe130 (CZ) | 3.77 |
| 23 | Arg250 (CD) | Gly4 (O) | 3.67 | 23 | Glu419 (OE1) | Phe130 (CE2) | 3.51 |
| 24 | Arg250 (CZ) | Gly4 (O) | 3.69 | 24 | Glu419 (OE2) | Pro129 (CA) | 3.6 |
| 25 | Arg250 (NH1) | Gly4 (C) | 3.58 | 25 | Glu419 (OE2) | Pro129 (C) | 3.27 |
| 26 | Arg250 (NH1) | Gly4 (O) | 2.88 | 26 | Glu419 (OE2) | Pro129 (O) | 2.71 |
| 27 | Arg250 (NH1) | Lys5 (O) | 3.55 | 27 | Glu419 (OE2) | Pro129 (CB) | 3.41 |
| 28 | Phe260 (CZ) | Phe9 (CD1) | 3.9 | 28 | Glu419 (OE2) | Phe130 (CG) | 3.87 |
| 29 | Phe260 (CZ) | Phe9 (CE1) | 3.88 | 29 | Glu419 (OE2) | Phe130 (CD2) | 3.35 |
| 30 | His267 (ND1) | CGU33 (OE22) | 3.46 | 30 | Glu419 (OE2) | Phe130 (CE1) | 3.77 |
| 31 | His267 (CE1) | CGU33 (CD1) | 3.43 | 31 | Glu419 (OE2) | Phe130 (CE2) | 3.01 |
| 32 | His267 (CE1) | CGU33 (CD2) | 3.36 | 32 | Glu419 (OE2) | Phe130 (CZ) | 3.25 |
| 33 | His267 (CE1) | CGU33 (OE11) | 3.86 | 33 | Ala457 (CB) | Thr144 (CB) | 3.83 |
| 34 | His267 (CE1) | CGU33 (OE12) | 3.21 | 34 | Ala457 (CB) | Thr144 (OG1) | 3.39 |
| 35 | His267 (CE1) | CGU33 (OE22) | 3.01 | 35 | Arg489 (CZ) | Thr35 (OG1) | 3.27 |
| 36 | His267 (NE2) | CGU33 (CD1) | 3.69 | 36 | Arg489 (NH1) | Thr35 (CB) | 3.59 |
| 37 | His267 (NE2) | CGU33 (CD2) | 3.47 | 37 | Arg489 (NH1) | Thr35 (OG1) | 2.86 |
| 38 | His267 (NE2) | CGU33 (OE11) | 3.7 | 38 | Arg489 (NH2) | Thr35 (OG1) | 3.62 |
| 39 | His267 (NE2) | CGU33 (OE12) | 3.83 | 39 | His534 (NE2) | Leu143 (CD2) | 3.36 |
| 40 | His267 (NE2) | CGU33 (OE21) | 3.57 | 40 | His538 (CE1) | CGU36 (CD2) | 3.82 |
| 41 | His267 (NE2) | CGU33 (OE22) | 3.47 | 41 | His538 (CE1) | CGU36 (OE22) | 3.04 |
| No. | FXIa-CD | FIXaβ-CD | Distance (Å) | 42 | His538 (NE2) | CGU36 (CB) | 3.6 |
| 1 | Pro392 (CB) | Ser283 (OG) | 3.66 | 43 | His538 (NE2) | CGU36 (CD2) | 3.89 |
| 2 | Pro392 (CB) | Tyr284 (CE2) | 3.66 | 44 | His538 (NE2) | CGU36 (OE22) | 3.38 |
| 3 | Pro392 (CG) | Ser283 (OG) | 3.31 | 45 | Lys539 (CE) | CGU36 (OE12) | 3.9 |
| 4 | Thr393 (OG1) | Glu242 (O) | 3.73 | 46 | Lys539 (NZ) | CGU36 (OE12) | 3.8 |
| 5 | Thr393 (OG1) | Thr244 (CG2) | 3.2 | 47 | Asp551 (OD2) | Arg145 (CZ) | 3.78 |
| 6 | Arg395 (CD) | Tyr284 (OH) | 3.24 | 48 | Asp551 (OD2) | Arg145 (NH1) | 3.78 |
| 7 | Arg395 (NE) | Tyr284 (OH) | 3.54 | 49 | Asp551 (OD2) | Arg145 (NH2) | 2.96 |
| 8 | Arg507 (CD) | Glu185 (CD) | 3.42 | 50 | Ala552 (C) | Arg145 (NH1) | 3.35 |
| 9 | Arg507 (CD) | Glu185 (OE1) | 3.46 | 51 | Ala552 (O) | Arg145 (CZ) | 3.46 |
| 10 | Arg507 (CD) | Glu185 (OE2) | 3.62 | 52 | Ala552 (O) | Arg145 (NH1) | 2.72 |
| 11 | Arg507 (NE) | Glu185 (CD) | 3.7 | 53 | Ala552 (O) | Arg145 (NH2) | 3.66 |
| 12 | Arg507 (NE) | Glu185 (OE1) | 3.3 | 54 | Cys553 (N) | Arg145 (NH1) | 3.73 |
| 13 | Arg507 (NE) | Tyr325 (OH) | 2.95 | 55 | Cys553 (CA) | Arg145 (NH1) | 3.59 |
| 14 | Arg507 (CZ) | Tyr325 (OH) | 3.39 | 56 | Lys554 (CA) | Arg145 (O) | 3.4 |
| 15 | Arg507 (NH2) | Tyr325 (OH) | 2.99 | 57 | Lys554 (C) | Arg145 (O) | 3.25 |
| 16 | Asp508 (O) | Lys188 (NZ) | 3.69 | 58 | Lys554 (O) | Arg145 (O) | 2.87 |
|  |  |  |  | 59 | Lys554 (NZ) | Gln139 (CA) | 3.83 |
|  |  |  |  | 60 | Lys554 (NZ) | Gln139 (CB) | 3.37 |
|  |  |  |  | 61 | Lys554 (NZ) | Thr144 (O) | 3.51 |
|  |  |  |  | 62 | Thr575 (CG2) | Arg145 (CG) | 3.82 |
|  |  |  |  | 63 | Thr575 (CG2) | Arg145 (NE) | 3.29 |
|  |  |  |  | 64 | Ser576 (C) | Arg145 (CG) | 3.61 |
|  |  |  |  | 65 | Ser576 (O) | Thr144 (OG1) | 3.36 |
|  |  |  |  | 66 | Ser576 (O) | Arg145 (N) | 3.54 |
|  |  |  |  | 67 | Ser576 (O) | Arg145 (CB) | 3.9 |
|  |  |  |  | 68 | Ser576 (O) | Arg145 (CG) | 3.24 |
|  |  |  |  | 69 | Trp577 (N) | Arg145 (CG) | 3.82 |
|  |  |  |  | 70 | Trp577 (N) | Arg145 (NE) | 3.76 |
|  |  |  |  | 71 | Trp577 (CA) | Arg145 (CG) | 3.68 |
|  |  |  |  | 72 | Trp577 (CA) | Arg145 (CD) | 3.85 |
|  |  |  |  | 73 | Trp577 (CA) | Arg145 (NE) | 3.38 |
|  |  |  |  | 74 | Trp577 (C) | Arg145 (NE) | 3.28 |
|  |  |  |  | 75 | Trp577 (C) | Arg145 (CZ) | 3.45 |
|  |  |  |  | 76 | Trp577 (C) | Arg145 (NH2) | 3.39 |
|  |  |  |  | 77 | Trp577 (O) | Arg145 (NE) | 3.67 |
|  |  |  |  | 78 | Trp577 (O) | Arg145 (CZ) | 3.69 |
|  |  |  |  | 79 | Trp577 (O) | Arg145 (NH2) | 3.19 |
|  |  |  |  | 80 | Trp577 (CB) | Leu143 (O) | 3.6 |
|  |  |  |  | 81 | Trp577 (CB) | Thr144 (CA) | 3.71 |
|  |  |  |  | 82 | Trp577 (CE3) | Leu143 (CG) | 3.75 |
|  |  |  |  | 83 | Trp577 (CE3) | Leu143 (CD1) | 3.61 |
|  |  |  |  | 84 | Trp577 (CZ3) | Leu143 (CD1) | 3.74 |
|  |  |  |  | 85 | Gly578 (N) | Arg145 (NE) | 3.51 |
|  |  |  |  | 86 | Gly578 (N) | Arg145 (CZ) | 3.34 |
|  |  |  |  | 87 | Gly578 (N) | Arg145 (NH1) | 3.8 |
|  |  |  |  | 88 | Gly578 (N) | Arg145 (NH2) | 3.45 |
|  |  |  |  | 89 | Gly578 (CA) | Arg145 (CZ) | 3.5 |
|  |  |  |  | 90 | Gly578 (CA) | Arg145 (NH1) | 3.74 |
|  |  |  |  | 91 | Gly578 (CA) | Arg145 (NH2) | 3.26 |
|  |  |  |  | 92 | Glu579 (CA) | Leu143 (CD1) | 3.59 |
|  |  |  |  | 93 | Glu579 (O) | Leu143 (O) | 3.77 |
|  |  |  |  | 94 | Glu579 (O) | Leu143 (CD1) | 3.78 |

**Supplementary Table S4.** Summary of specific non-covalent inter-domain contacts maintaining the resting conformation of the FXI-CD within FXI using available zymogen FXI crystal structure (PDB 6I58)^17^.

| **Zymogen FXI** | |
| --- | --- |
| **I. Catalytic Domain Anchoring via the A2-A3 Linker** | |
|  | **Asp488, Cys267, Leu262, Ile269 🡪 Arg184** |
|  | **Leu601, Asp598 🡪 Leu177** |
|  | **Lys486 🡪 Asn179** |
|  | **Gly487 🡪 Ala181** |
| **II. CD Restraint via the A3-A4 Linker** | |
|  | **Val568 🡪 Ser268, Pro270, Ile269** |
| **III. Pre-cleavage AP Engagement** | |
|  | **Gly379, Asp476, Arg479 🡪 Arg369 (P1 Arg)** |
| **IV. AP-mediated Trans-Domain Bridging** | |
|  | **Tyr474 🡪 Glu361 (AP)** |
|  | **Glu361 (AP) 🡪 Gln88 (A1-A2 Linker)** |
|  | **Lys365 (AP) 🡪 Ser275 (Apple 4)** |

**Legends for supplementary videos**

Supplementary Video S1. Cryo-EM map and atomic model of the full-length FXIa-FIX complex. A 90-degree rocking view of the three-dimensional cryo-EM density map overlaid with the refined atomic model of the FXIa:FIX complex.

**Supplementary Video S2. Cryo-EM map and atomic model of the full-length FXIa-FIXa**$\boldsymbol{\beta}$ **complex.** A 90-degree rocking view of the three-dimensional cryo-EM density map overlaid with the refined atomic model of the FXIa-FIXa$\beta$ complex.

**Supplementary Video S3. FIXa**$\boldsymbol{\beta}$ **Catalytic domain.** A focused 360-degree rotation of three-dimensional cryo-EM density map corresponding to the FIXa$\beta$ catalytic domain region of the FXIa-FIXa$\beta$ complex overlaid with the refined atomic model of the FIXa$\beta$ in gold. Residue Val181 is highlighted in green and Asp364 is highlighted red.

**Supplementary Video S4. 3D Flex analysis of the FXIa:FIXa**$\boldsymbol{\beta}$ **complex.** A movie generated using 3D Flex analysis in cryoSPARC illustrating the continuous conformational dynamics of FIXa$\beta$-CD. The movie tracks the flexible movement of the FIXa$\beta$-CD (as well as EGF domains) as it hovers relative to the rigid FIXa$\beta$-Gla, FXIa-CD and FXIa-A3 exosite.

**Supplementary Video S5. FXI activation-associated functional conformational change.** Morph movie depicting the transition of the catalytic domain (magenta) from zymogen state in FXI (cyan, PDB 6I58), to the active state in FXIa (gold). ChimeraX-defined principal axis through the catalytic domain is shown in green for the zymogen and blue for FXIa. These axes were used to measure the pivoting angle of the CD.
